# Developing an emotional coping skills workbook for inpatient psychiatric settings: a focus group investigation

**DOI:** 10.1186/s12888-018-1790-z

**Published:** 2018-06-22

**Authors:** Molly Sharp, Anu Gulati, Chris Barker, Kirsten Barnicot

**Affiliations:** 1 Department of Clinical, Educational and Health Psychology, University College London, Gower Street, London, WC1E 6BT UK; 2 Centre for Psychiatry, Department of Medicine, Imperial College London, Commonwealth Building, Du Cane Road, London, W12 0NN UK

**Keywords:** Focus groups, Inpatients, Mental health, Psychiatric hospitals, Psychosocial intervention, Qualitative research, Thematic analysis

## Abstract

**Background:**

Evidence suggests an unmet need for provision of psychological interventions in inpatient psychiatric settings. However, inpatient wards can present a challenging environment in which to implement interventions. The authors developed the Emotional Coping Skills workbook, a psychosocial intervention designed to overcome these challenges and provide inpatients with an opportunity for psychologically-informed therapeutic engagement. The workbook includes information and exercises to empower inpatients to understand their emotions and learn to cope with their distress.

**Methods:**

A qualitative study using thematic analysis was undertaken in two UK inpatient psychiatric hospitals to explore staff’s views about whether and how the workbook could be implemented, and on barriers to its use. Thirty-five nursing and occupational therapy staff members participated in four focus groups, and a further two psychologists in semi-structured interviews.

**Results:**

Staff identified key barriers to successful implementation of the workbook. These were firstly, the difficulty in finding time and space for therapeutic work in the stressful ward environment. Secondly, staff identified a culture of emotional neglect whereby neither staff nor inpatients felt able to talk about emotions, and patients’ physical needs and medication were prioritised. Thirdly, staff discussed how psychotic symptoms and emotional distress could limit patients’ ability to engage with the workbook material. Staff suggested ways in which the feasibility of using the workbook could be enhanced. Firstly, they discussed the importance of encouraging staff to value psychological approaches and to view the workbook as a resource to help them manage their existing tasks. Secondly, they emphasised the value of staff drawing on their expertise to deliver the workbook flexibly in different formats and settings, depending on each patient’s particular presentation. Thirdly, they advocated empowering staff to decide the timing of intervention delivery in the context of each inpatient’s fluctuations in distress and progress towards recovery.

**Conclusions:**

The study has highlighted key principles for flexible and well-integrated intervention delivery; these principles will be helpful for enhancing the feasibility of any nurse-delivered psychological intervention in inpatient settings.

## Background

There is a growing impetus to increase access to psychologically-informed interventions in inpatient psychiatric settings. The Care Quality Commission [1] revealed that half of inpatients surveyed wanted access to psychological therapy. A report by the Commission on Acute Adult Psychiatric Care stated that UK inpatients and carers desire a wider range of therapies to be made available to inpatients, including psychological therapies [2]. The report emphasised the need for acute mental health services to “deliver a full range of evidence-based biopsychosocial and physical interventions which focus on the patient’s recovery” (p.57). Despite these recommendations, nursing staff spend a disproportionately small amount of time conducting psychological interventions than social, physical or pharmacological ones [3]. Whilst inpatient staff teams are characterised by multidisciplinary work, nursing staff are involved in the social milieu of the ward to a different extent to other health professionals [4], and frequently encounter inpatients’ intense distress. Although nursing staff spend an average of 50% of time in contact with inpatients, just 4–20% of this involves working therapeutically [5].

The inpatient psychiatric ward is a particularly difficult environment in which to deliver psychological interventions. Firstly, individuals meeting the threshold for admission to inpatient care are characterised by acute mental health crises, which may manifest in extreme emotional distress, impulsive self-damaging behaviour or aggression towards others, severe psychotic symptoms, and impairments in concentration and attention [2, 6–8]. These presentations may make it difficult for psychiatric inpatients to engage with traditional psychotherapy [9]. Secondly, the length of inpatient stay in the UK is often brief. The Health and Social Care Information Centre state that the median length of stay for people discharged from inpatient mental health services in 2013–2014 was just 23 days [10]. This limits the time available to build a therapeutic relationship, formulate an adequate understanding of the inpatient’s psychological difficulties, and conduct therapy sessions [9, 11]. Thirdly, high levels of demand for acute services mean that staff are under pressure to release beds and tackle administrative duties whilst handling inpatient crises involving high levels of risk [2, 12–14]. In the context of staff shortages [15], this contributes to excessive emotional exhaustion and work-related ‘burnout’ [16, 17]. Staff may often feel that they lack the time, or emotional energy, to deliver psychological interventions.

Aarons and Sawitzky [18] propose that the uptake of evidence-based interventions in mental health settings depends on the setting’s organisational climate and culture. The factors discussed above contribute to the complex organisational structure of inpatient psychiatric hospitals. An inpatient population characterised by acute psychological distress, short and highly variable hospital stays, and pressurised services mean that these environments are dominated by risk-oriented practice. Aarons and Sawitzky [18] describe defensive organisational cultures as those that: “encourage or implicitly require interaction with others in ways that are self-protective and will not threaten perceived personal security” (p.62). Successful risk management is vital in inpatient psychiatric care, and yet emphasis on this may pose a significant challenge to encouraging staff to implement psychological interventions [19], by contributing to a defensive organisational culture that is “resistant to change” [4, 20].

High levels of emotional distress, coupled with difficulties in self-regulating, are a common experience amongst psychiatric inpatients, irrespective of their clinical diagnoses [7]. The authors therefore developed a psychosocial intervention that could empower inpatients to understand their emotional distress and to develop coping strategies. The content and modes of delivery utilised were intended to bypass the challenges discussed in implementing psychological interventions in inpatient settings. Inspired by Clarke and Wilson’s work on this topic [9], the authors drew on ideas from Dialectical Behaviour Therapy (DBT) [21], and Cognitive Behavioural Therapy (CBT) [22] to inform the intervention content. DBT asserts that emotional distress is maintained by invalidation of one’s emotions by oneself and the social environment, and the lack of necessary skills to self-regulate [23]. CBT suggests that emotional distress is maintained by cognitive distortions or ‘thinking errors’ [22]. Previous studies have demonstrated the benefits of using both DBT and CBT in inpatient settings. However, such studies have typically involved specialist residential units, diagnosis-specific groups, intensive formats such as daily group sessions, or time periods that exceed the typically short length of stay in an acute psychiatric ward [10, 24–27]. The authors wished to harness these therapeutic techniques to create a more flexible and generalisable intervention for typical inpatient care settings.

The developed intervention is a workbook that contains two sections. Part One aims to help inpatients develop a better understanding of their emotions. Firstly, inpatients are asked to focus on a recent episode of emotional distress. They are encouraged to try to use ‘emotion words’ to label their feelings, and they identify the events and thoughts that preceded an increase in emotional distress, the bodily manifestations of the emotion (e.g. sweating, heavy chest, heart racing) and any behavioural urges precipitated by the emotions. Part Two of the workbook aims to teach inpatients about coping strategies for regulating their emotions, such as challenging their thoughts and interpretations of a distressing situation or using a variety of distraction techniques.

The authors sought to develop intervention content and modes of delivery that could overcome some of the challenges to implementing psychological interventions in inpatient settings, by:

1. Presenting information that is applicable across a wide range of clinical diagnoses. This was addressed by focusing on the trans-diagnostically relevant concept of learning to understand and cope with emotional distress.
2. Using a workbook format that could be applied by nursing staff in their everyday interactions with inpatients, in order to create an intervention that nurses could deliver without increasing their workload. Nursing staff could use the workbook during daily scheduled one-to-one time with their allocated caseload of inpatients. The workbook was designed to be self-explanatory, requiring minimal staff training.
3. Communicating information using simple messages illustrated with pictures, to ensure accessibility for acutely distressed inpatients with impairments in attention and memory.
4. Facilitating inpatients to apply the information to their individual situations as often as needed and to develop a therapeutic rapport with the nurse delivering the workbook. The workbook was designed to be delivered in a one-to-one format by an inpatient’s allocated staff nurse rather than during group sessions.
5. Teaching coping strategies that are applicable and workable in the restricted environment of an inpatient psychiatric unit.

Following preliminary development of the intervention, the authors aimed to conduct a qualitative focus group investigation of the views of nursing staff and allied mental health professionals on the potential benefits, negative effects, feasibility and barriers to using it. This is in line with Medical Research Council [28], British Medical Journal [29] and National Institute of Clinical Excellence [30] guidance on the development of complex interventions, which state that early-stage development should use qualitative methodology to explore the views of workers intended to implement an intervention about its workability and barriers [28]. By contrast, it has been argued that top-down approaches to introducing new interventions into health services that do not incorporate the views of frontline staff contribute to unsuccessful intervention implementation [19]. It is also in line with the tenets of Normalisation Process Theory [31] - a theory that delineates factors that promote and inhibit the routine incorporation of complex interventions into everyday practice. In evaluating whether an intervention can feasibly be incorporated into routine practice, NPT asks whether the intervention is coherent (can clinicians differentiate it from their normal clinical practice; is it believed it will be valuable; and will it fit with the values of the investigation). It also asks what cognitive participation will be like (will participants engage and commit to the intervention). Furthermore, NPT considers what the collective effect of the intervention will be (will it help or impede existing working practice; is extensive training required) [32]. NPT emphasises the importance of considering the context of where the intervention will be deployed during the process of development, and to examine staff’s current and foreseeable concerns to assess whether the proposed intervention will fit [32].

The present investigation aimed to assess whether, in the views of inpatient psychiatric staff, the workbook intervention could be a valid method of addressing inpatients’ emotional distress, and whether they believe its implementation to be feasible in inpatient psychiatric settings, by addressing the following research questions:

1. What is the opinion of inpatient psychiatric staff about any potential benefits or negative consequences of using the workbook with inpatients?
2. To what extent does the workbook fit in with already existing methods used by staff to manage inpatients’ emotions?
3. What barriers do staff envisage in using the workbook, and in what ways could it be improved?

## Methods

### Research design

This study is an applied qualitative research investigation of the opinions of inpatient psychiatric nursing staff and allied mental health professionals on the workbook’s usability and feasibility. The Medical Research Council (MRC) and the British Medical Journal (BMJ) recommend incorporating qualitative research to develop complex interventions, particularly when identifying potential barriers [28, 29]. Qualitative methodology produces rich data and enables the exploration of complex aspects of experience that may be oversimplified by quantitative approaches [33].

Communication between research participants in a focus group study allows them to explore and clarify ideas in a way that is less easy to do in one-to-one interviews [34]. NICE advocate using focus groups to identify potential barriers in implementing new interventions [30]. Accordingly, the study employed a focus group investigation. In addition, two ward psychologists were interviewed individually, to triangulate and enrich the data.

### Research setting

The investigation was carried out on the acute admission, recovery, and intensive care (PICU) inpatient wards of two inner-city psychiatric hospitals in the UK.

### Focus group participants

Focus groups were conducted with nursing staff and allied mental health professionals in order to gain feedback on the usability and feasibility of the workbook. Whilst typified by multidisciplinary work, nursing staff are frontline workers in inpatient psychiatric hospitals [35]. They are primarily involved in implementing any intervention and can therefore be considered key “stakeholders” [28]; thus, they predominated the sample. A multitude of other mental health professionals work within inpatient psychiatric teams, and their support would also be vital to the success of implementing a novel intervention. In order to ensure these perspectives were considered, any nursing staff, occupational therapists or other inpatient mental health professionals currently working on an inpatient psychiatric ward were eligible to participate.

Staff attendance at focus groups was organised by ward managers on the basis of staff availability and level of qualification. Separate focus groups were conducted with unqualified/ recently qualified staff (NHS Band 5 and below) and with more senior staff (NHS Band 6 and above). The separation of unqualified/recently qualified staff from senior staff aimed to minimise the influence of hierarchy during discussion. Four focus groups, each with 6–12 participants, were conducted in order to ensure sufficient group members to draw out conflicting points of view and generate discussion, whilst allowing each person to talk in adequate detail about their point of view – a balance between quantity and quality [36].

### Focus group procedure

The focus groups were co-facilitated by study authors KB, MS and AG. Participants were first provided with an information sheet about the study and an opportunity to ask questions. Next, they provided informed consent and socioeconomic information. Following this, participants were given a copy of the workbook, and the facilitators used a semi-structured topic guide to direct discussion of the workbook’s potential benefits, barriers to its use, and suggested improvements. Participants were asked open-ended questions pertaining to these areas of interest. The topic guide included examples of typical scenarios that might occur on the ward, used to help the participants to imagine using the workbook with inpatients in their day-to-day work. Before commencing the discussion, participants were asked to be respectful of the opinions of others, and the facilitators emphasised the importance of hearing from every group member. Each focus group lasted approximately 1 h and was audio-recorded for later transcription.

### Individual interviews

Using the same topic guide, author MS conducted individual interviews with two ward psychologists employed at each hospital, in order to triangulate the focus group data and provide a broader perspective on the intervention’s usability and feasibility.

### Analysis

A critical realist approach was adopted towards the data, recognising the interplay between social structure and human agency, and the effect this has on the research conducted [37]. Thematic analysis was conducted using QSR NVivo data analysis software [38], applying an inductive approach to code generation, and following Braun and Clarke’s [39] guidelines on conducting thematic analysis. Following generation of preliminary codes, the coding framework was refined by adding, removing or combining codes in order to maximise internal homogeneity and external heterogeneity, and codes were finally grouped into over-arching themes and sub-themes [39]. Analysis was led by author MS and regularly reviewed and refined by co-authors KB and AG, with supervisory oversight of the coding framework by CB. This team-based approach to coding intended to increase the credibility of the derived themes [40].

## Results

### Description of the sample

Thirty-seven staff members agreed to participate. Their characteristics are summarised in Table 1. The sample included thirty-two nursing staff (86.48%) in addition to occupational therapists and psychologists. Sixteen of the sample were employed within NHS band 5 or under (and took part in the two focus groups comprised of more junior staff) and 19 were employed at band 6 and above (and took part in the two focus groups comprised of more senior staff). Twenty-two of the participants identified as women (59.45%), and the mean age of the sample was 41 (range = 24–62). The sample included participants with a range of ethnic identities (70.27% Black, Asian and Minority Ethnic). The participants spanned an array of staff roles and years spent working in inpatient mental health care.

**Table 1:** Participant characteristics

		Focus Group 1	Focus Group 2	Focus Group 3	Focus Group 4	One-to-One Interviews
Total N		6	10	8	11	2
Hospital		1	2	2	1	1 & 2
Banding (NHS Afc ^a^)		≤ 5	≤ 5	≥ 6	≥ 6	≥ 6
Gender, N (%)	Men	5 (83)	3 (30)	2 (25)	5 (45)	N/a
	Women	1 (17)	7 (70)	6 (75)	6 (55)	2 (100)
Ethnicity, N (%)	White	2 (33)	3 (30)	3 (38)	1 (9)	2 (100)
	BAME	4 (67)	7 (70)	5 (62)	10 (91)	N/a
Staff role, N (%)	Unqualified nursing staff	4 (67)	6 (60)	N/a	N/a	N/a
	Qualified nursing staff	2 (33)	3 (30)	N/a	2 (8)	N/a
	Occupational therapist	N/a	1 (10)	2 (25)	N/a	N/a
	Senior nursing staff ^b^	N/a	N/a	6 (75)	9 (82)	N/a
	Clinical psychologist	N/a	N/a	N/a	N/a	2 (100)
Years experience working in inpatient mental health care, M (SD)		5.7 (3.7)	4.8 (6.1)	8.6 (3.9)	11.4 (6.4)	–

^a^NHS Job Evaluation Handbook (2016), NHS Terms and Conditions of Service (2017)

^b^Includes clinical team nursing leads and ward manager

### Thematic analysis

Thematic analysis of the focus groups and interviews yielded three central themes characterising staff views on the extent to which the workbook is a good fit with current nursing practice and the ward environment (Theme 1), and on how (Theme 2) and when (Theme 3) the workbook could be used.

#### Theme 1 The ‘fit’ of the workbook

How far the workbook intervention integrates with nursing staff practices and the ward environment more generally was discussed at length. This feedback was organised into three sub-themes: (1.1) A stressful environment, (1.2) A culture of emotional neglect and (1.3) Adding structure and confidence to current nursing practice. Specific quotes given by participants on this theme can be found in Table 2.

**Table 2:** Examples of participant quotes for theme 1 The ‘Fit’ of the workbook

1.1 A stressful working environment	FG3-P7: Coming to them with more paperwork is just like ‘Are you kidding me?’ It’s like asking for […] a limb. FG1-P1: In a ward where things happen so quickly, you might not have the time to be able to sit with the patient and actually go through this. FG2-P2: Recently we had one, she actually broke the door …..the team are all trying to deal with her but she had broken the fire and the doors are wide open and she managed to leave. I2: The institution has to be run to keep the institution running and keep the people safe – it’s not centred on the needs of the people who are in it. FG3-P7: I think it’s how it’s sold to them. And how you sit and talk through and say, you know, ‘you do provide one-to-one to your patient, all’s we’re asking is that you bring this book… I think it’s my job and it’s [my clinical team leader’s] job to stand there and go ‘We need to stop and think why exactly why are we here - we are here for the patients and to provide them one-to-one time.’
1.2 A culture of emotional neglect	FG4-P6: Some of our patients we have to forcibly give IM medication, we have to put them in seclusion….You’re chemically calming someone down with medication. I2: The hospital is very medical though, in that it is symptom-focused, it’s not emotion-focused… there often isn’t an interest in the phenomenology of emotion because it isn’t really part of the medical model… [it’s] about stabilising people medically and getting them back into the community. FG3-P3: We witness lots and lots of…distress symptoms and emotions from people …some staff that don’t have, necessarily, the confidence – will feel very resistant. I think that they’re going to be quite scared of what people might say and then how to deal with that. FG2-P1: We’ve tried to …get patients to reflect just recently about small, superficial self-harming behaviours, and they don’t want to make the connections with their emotions. They’re just demanding …cigarettes and medication. They don’t want to look inwards at their emotions FG3-P3: A lot of people who come through our doors haven’t actually gained those words in their youth….What are emotions? What actually are the emotions you experience? Therefore they can’t describe them because they don’t know them.
1.3 Adding structure and confidence to current nursing practice	FG1-P4: A few things that you point out [are] what we do already anyway…. things like trying to encourage patients to try and distract themselves ….And other situations where we’ve tried to sit down with the patients and identify why they got so emotionally aroused in the first place. FG3-P7: For their one-to-ones it’s definitely something they’re [already] doing…. this is just more formally put on paper, it’s structured and if you’re sitting with somebody with this in front of you it feels more like an engagement, it just seems more therapeutic. FG1-P5: This book is very informative, educative, there is a lot of useful information that we can actually deduce from this book. It actually refreshes what you know…it’s very helpful. FG1-P1: The questions in there to ask would be quite helpful [for]…trying to get them to use their own insight to figure out why they feel like this. FG2-P2: I think it’s good to read through to get ideas for engagement tactics …it helps me understand my clients better and then …gives me ideas for more positive interactions I can have with my patients.

##### Sub-theme 1.1 A stressful environment

Participants highlighted that the ward environment could present an impediment to implementing the workbook. They repeatedly remarked that wards were under-resourced, and staff were consequently over-worked. Most believed this would impede use of the workbook, as staff might consider it an extra duty and burden. Participants emphasised that the primary role of ward staff was to maintain safety and reduce risk. Understaffing could necessitate the whole staff team’s involvement in managing a serious event, reducing the opportunities for therapeutic work with other inpatients.

One psychologist felt that staff’s duty to ensure the safety of the ward as a whole overshadowed the emotional needs of specific inpatients. Participants in the focus groups with senior staff acknowledged these potential difficulties, but ward managers in particular felt it was their responsibility to present it in such a way that their staff would feel encouraged and not burdened by being asked to use it. They suggested emphasising that the workbook could add structure to what staff already do with inpatients, help staff get to know their inpatients better and facilitate their recovery, and help them to formulate care plans more easily.

##### Sub-theme 1.2 A culture of emotional neglect

Participants’ accounts suggested that a culture of emotional neglect could present an impediment to implementing the workbook. Staff’s explanations of their current methods of managing emotional distress reflected a highly medicalised perspective. In all the focus groups, medication, including pro re nata and forcibly administered intramuscular injections, was described as a primary method to manage inpatient distress. One psychologist proposed that the perceived superiority of the medical framework encouraged nurses to view inpatients’ emotions only in relation to their symptoms, which could hinder use of the workbook. Relatedly, senior staff felt some of the less experienced staff would lack the confidence to engage in therapeutic work and could worry about how to respond to inpatients’ distress. Participants described inpatients as often being unable or unwilling to discuss their emotions and attributed this to a lack of effective ways of understanding and articulating negative feelings. Additionally, staff felt that, rather than wanting to discuss their emotional needs, inpatients tended to be insistent on coping with their distress using physical means such as medication and smoking, as these provided instant gratification. This tendency to seek instant gratification rather than engage in discussions about emotions might obstruct use of the workbook.

##### Sub-theme 1.3 Adding structure and confidence to current nursing practice

Staff concurred that the workbook intervention could be a valid resource for helping to ameliorate inpatients’ emotional distress. Many participants felt that staff already used some of the ideas in the workbook during their everyday interactions with inpatients. They felt that the workbook could help them to better structure their conversations, make their one-to-one sessions with inpatients more meaningful, and make formulating care plans easier. Staff anticipated that having a resource to draw on would bolster staff confidence and, for more experienced staff, refresh memories and reinforce confidence in their therapeutic work. They believed that it could provide new ideas about how to engage inpatients in discussing their emotions. Furthermore, participants felt the workbook could provide inpatients with a device to help articulate their emotions and could help staff to better understand and empathise with inpatients.

#### Theme 2 Conveying the workbook

Participants suggested ways in which the contents (sub-theme 2.1) and mode of delivery (sub-theme 2.2) of the workbook could be optimised in order to overcome barriers to its implementation. Specific participant quotes relating to this theme can be found in Table 3.

**Table 3:** Examples of participant quotes for theme 2 Conveying the workbook

2.1 Optimising the content	I2: The thoughts that people have when they’re psychotic ….have that utter ring of truth… you’d have to begin by saying ‘I know that feels 100% true, but let’s just have another hypothesis’ (I2). FG4-P6: Our feelings always make sense on one level or another’. It’s quite an interesting phrase really; if you’ve got delusional beliefs that you’re Jesus, God, da Vinci, whoever, now…uh…to sort of say ‘That makes sense’…. I’m uncomfortable with that actually. FG2-P5: Some of them, their concentration levels are…just below there. So, to have them concentrate on something is to have something which has got an impact on them, like a picture. Bright coloured picture. FG2-P1: They’re very visually orientated with things …. [whereas with the] amount of content on the page - they might just go ‘Well you can skip that right out!’
2.2 Delivering the content	FG1-P1: If I went in there with one of these and tried to say ‘Let’s look at your feelings’…they’d say ‘Leave me alone for now’…if I went in there…like ‘Let’s talk about some of the things that’s going on in your life that’s distressing you’…you bring emotion into it along with what the problems are. So it’s a different way of bringing in the emotion. I1: So it’s something about making it visible? And…not very stigmatised, in terms of you know, you could pick up a magazine, you could pick up this. Just so that it’s part of the culture really…so the patients have got it and it’s around. FG4-P4: For me, I feel this book also you can’t implement it as a whole, but each part has its importance, and I think some of our nurses and our support workers…they can take part of it and do that one-to-one, about your feelings, about…‘What made you sad?’, and…use that book as a tool. FG1-P2: Maybe like a session on...a summary of physical traits of different emotions…I think if you...went and gave someone this they’d just be like ‘Oh gosh, what’s all this?’ But I think if you just…take it down into bite-sized chunks.

##### Sub-theme 2.1 Optimising the content

Some participants felt that the workbook could be adapted to fit a greater variety of clinical presentations, suggesting it was geared towards inpatients with personality or affective disorders, and less applicable to those with psychotic disorders. They proposed that the workbook's emphasis on encouraging inpatients to challenge their thoughts could be particularly difficult for psychotic inpatients that are convinced that their thoughts reflect reality. By contrast, others felt that the first half’s emphasis on validating inpatients’ feelings as ‘making sense’ could be misinterpreted by psychotic inpatients as staff corroborating their often distressing ‘delusions’.

Positive feedback was given regarding the workbook’s simple language and lack of jargon, use of large, bold letters and the inclusion of pictures. However, some participants worried that inpatients’ comprehension level and concentration span might nevertheless impede the workbook’s usefulness. They considered that including even more pictures and colours with less text could help to overcome this.

##### Sub-theme 2.2 Delivering the content

Participants debated how best to utilise the workbook whilst minimising the possibility of exacerbating inpatient distress and maximising the chance that inpatients would engage in conversations about emotions. Some participants felt that inpatients were more likely to talk about their emotions if the topic was at first approached indirectly in the context of a more general conversation about their wellbeing, rather than by launching into the workbook straight away.

Participants discussed the availability of the workbook in the ward. Some said copies of the workbook should be left in communal spaces, such as the lounge and TV room, and that it could be promoted via posters on notice boards and leaflets. They felt that making the workbook available in as many locations and formats as possible would help to get inpatients interested in looking at it on their own or with staff and encourage embedding of the workbook into the culture of the ward.

Participants debated whether staff should help inpatients to use the workbook or whether inpatients could use it as a self-help resource. Some felt that in certain circumstances inpatients could use the workbook alone, but its usefulness in this context was said to depend on the characteristics of the individual inpatient, such as their curiosity, education-level and the acuity of their clinical presentation. In all of the focus groups and both interviews, participants believed that it would be helpful for staff to assist inpatients in using the workbook. The workbook was deemed particularly appropriate for one-to-one inpatient-staff interactions. Many participants suggested that breaking the workbook down and using small sections of it at a time would make it more manageable for staff and inpatients. Some suggested that principles from the workbook could be abstracted and used in conversation with inpatients, without necessarily presenting the workbook. There were suggestions of workbook pages being turned into posters for the wards, or one page at a time being used for a ward group activity.

#### Theme 3 Getting the timing right

Staff emphasised that thinking carefully about the appropriate time to use the workbook with inpatients would be crucial to its success in being able to address inpatients’ emotional distress. This meant thinking about appropriate timing in the context of an inpatients’ overall stay in hospital (sub-theme 3.1), and in the context of individual inpatients’ hour-by-hour fluctuations in levels of emotional distress (sub-theme 3.2). Specific participant quotes on this theme can be viewed in Table 4.

**Table 4:** Examples of participant quotes for theme 3 Getting the timing right

3.1 A gradual trajectory towards recovery	FG3-P6: They might be too unwell to even sit down and engage with you and …let alone take a book and try to understand what they’re feeling. FG2-P5: They might not even understand what you are saying. ….Somebody that is psychotic - you are telling the person to go and watch TV, and the person is telling you that somebody is talking to him or her through the TV. FG3-P8: If you just have the booklet and …we go through as much as we can on the assessment wards and then once they are transferred to recovery they can then pick up where we’ve left off. FG3-P7: I think there is some patients you get …that just would not engage at that time that they’re on assessment, but once they are transferred, let’s say they do go to recovery, they can continue to try and…d’you know encourage somebody to engage in that. FG3-P3: It’s about knowing that we work with people that don’t necessarily engage, we just continue to try….. And that’s the best that we can do at this present moment. And it might not be for weeks before they’re actually at a place …to even explore anything like this.
3.2 The eye of the storm	FG2-P2: I think maybe giving someone this in the middle of when they’re like crying their eyes out, really, really distressed is probably not gonna do much initially… FG1-P1: But patients on PICU - sometimes they are very settled and stable but maybe the next day or in a few hours, they’d be like that. So, if you can catch them in a stable moment and use it then and maybe you can sow the seeds that might work then. FG3-P6: You can adopt it after that initial…d’you know, crisis period. […]when you’re reflecting back on what happened: ‘what led to that?’, ‘how are you feeling now?’ and then go through it. FG1-P1: That’s the main focus isn’t it, getting the glass off them, getting the instrument that they’re using…so, to bring out the book, I’m afraid, would be completely the wrong timing. Yeah, it’s all about timing.

##### Sub-theme 3.1 A gradual trajectory towards recovery

A barrier to the workbook discussed at length was the acuteness of inpatients’ presentations in the early stages of admission. Participants said that people in a highly acute stage of presentation had low levels of attention and concentration, which would impede use of the workbook, particularly when coupled with the ‘delusions’ characteristic of acute psychosis.

Some staff felt that using the workbook would be too difficult on assessment wards, where inpatients tend to be more acutely unwell. Others believed that it could be used on assessment wards with inpatients who have less acute presentations. One group reached consensus that the workbook could be used flexibly; introduced at assessment and followed-up in recovery when the inpatient was improving.

##### Sub-theme 3.2 The eye of the storm

Participants commented that within their overall trajectory towards recovery, inpatients could experience fluctuating levels of emotional distress on an on-going basis. They frequently discussed whether the workbook could be applied in moments of extreme distress, with many feeling it would be best to wait until the inpatient had calmed down a few minutes or hours later. Participants hoped that if the workbook was used with inpatients at calm moments, they might apply the skills and strategies acquired when future crises arose. Staff also emphasised that, if inpatients were engaging in risky behaviour such as self-harm or aggression towards others, taking practical measures to ensure the safety of staff and inpatients must be prioritised above discussing the inpatient’s emotions.

## Discussion

### Main findings

The present study aimed to explore opinions of inpatient psychiatric nursing staff regarding the validity, feasibility and acceptability of a workbook designed to help inpatients understand and cope with emotional distress. Staff felt the workbook was a valuable resource that could increase their confidence in addressing inpatients’ emotional distress and help them to structure their conversations around this topic. They suggested the validity of the workbook could be improved by ensuring it the content was relevant across all diagnostic presentations, and that this would help with implementation. They emphasised the value of creating a resource that could be flexibly used in different formats. Furthermore, they discussed the necessity of thinking carefully about the right time to introduce the workbook, depending on the individual inpatient’s hour-by-hour fluctuations in emotional distress, and what stage they are at within their overall recovery journey.

### Implications for implementing psychological interventions in inpatient psychiatric settings

Staff emphasised the importance of introducing the workbook at times when inpatients were feeling calmer, rather than in moments of acute distress. Similarly, they felt that using the workbook with inpatients at an acute stage of presentation would be ineffective, as they would not be able to understand or concentrate on the material. They suggested it would be more helpful for inpatients who were further along in their journey towards recovery. This has wider implications for the timing and nature of psychological interventions on inpatient wards, suggesting that interventions are more likely to be effective and incorporated into practice when staff are able to adjust the timing of their delivery in the context of each inpatient’s fluctuations in distress and overall stage of recovery. This could be more effective for individual inpatients than, for example, a rigid schedule of weekly sessions that does not adapt to the individual inpatient’s needs. Nursing staff, who are with inpatients at all times and observe changes in their mental state on a day-to-day basis, may be best placed to implement such a flexibly timed intervention [19].

Staff also suggested that additional work was needed to validate the workbook content to ensure it was relevant for inpatients with psychotic presentations. In particular, the sections on helping inpatients to recognise and challenge the patterns of thoughts contributing to their emotional distress could be adapted to draw on ideas from CBT for psychosis [41]. This might help to increase the focus on validating inpatients’ feelings in response to ‘delusional’ thoughts, whilst enabling them to consider the evidence for their thoughts. The lack of emphasis on psychotic presentations in the initial version of the workbook may reflect the difficulty of attempting to create a cross-diagnostic resource whilst drawing ideas from diagnostic-specific therapeutic models such as DBT. This therapy was primarily developed for individuals who were chronically self-harming, and those meeting criteria for emotionally unstable personality difficulties [21].

The stressful working environment of inpatient wards and the dominant emphasis on risk management and medical models of mental illness were also described as key barriers to implementing the workbook. It is well established that the inpatient psychiatric hospital is a stressful working environment [12, 16]. As reported elsewhere, staff in the present study explained that difficulties with understaffing and an often-chaotic ward environment could limit opportunities for therapeutic work [2, 12, 13, 19]. Participants worried that the workbook could be perceived as an extra burden amongst the large amounts of administration staff are required to complete. Applying NPT, this worry might lead to low cognitive participation with the intervention, which could result in low collective action (an unwillingness to invest time or energy in implementation) [32]. To counter this, staff stressed that being able to break the workbook down into smaller sections could make it easier for both staff and inpatients to manage. This suggests that allowing staff to flexibly apply the parts of interventions they feel are most appropriate for each inpatient could increase the utility of those interventions. Indeed, in their analysis of staff fidelity of different behavioural interventions, McConnachie and Carr [42] suggest that ‘user friendliness’ of treatment protocols might be critical for successful treatment implementation. Furthermore, when new psychological interventions are introduced it is vital to acknowledge the stressful working climate. The intervention should be emphasised as a tool that could help staff by making what they already do with inpatients easier [19], such as providing additional structure to one-to-one time already spent with inpatients and generating useful information to incorporate into care plans. Participants also commented on the workbook’s potential to help them understand their inpatients and assist in developing strategies to handle and avert future crises. Focusing on the ways in which the intervention is coherent with the needs and concerns of staff might help to foster cognitive participation and collective action regarding use of the intervention [32]. Indeed, the ‘fit’ of new interventions with the existing environment, skills, and capacity of those enforcing it has been identified as key for successful implementation [43].

Some participants felt that the dominant emphasis on risk management and medical models of mental illness within inpatient wards, in addition to inpatients’ reluctance to directly address their emotional difficulties, could often lead to emotional issues being neglected. Linked to this, it has been argued that an emphasis on medicalisation of psychiatric diagnosis can lead to pathologising human distress without understanding its context, neglecting to address the interpersonal trauma and social disadvantages that in many cases contribute to psychological distress [44]. Related to this culture of emotional neglect, inpatients were reported to frequently be unwilling or unable to talk about their emotions and to instead focus on resolution of their distress by practical means such as cigarettes and medication. Participants reported the central use of medical and physical management approaches by staff, adding to literature suggesting that a medical framework still dominates inpatient psychiatric practice [9, 45] whilst psychosocial therapeutic strategies remain underused [3, 5, 46]. Participants worried that staff might feel under-confident and inexperienced in discussing emotional issues with inpatients. This suggests that training for staff in implementing psychological interventions should emphasise the importance and relevance of psychological approaches to emotional distress, and how these approaches might benefit their work. In the context of the workbook, we would hope to reiterate the coherence of the intervention with staff’s needs. Specifically, we would aim to elucidate the ways in which therapeutic engagement with emotional distress might help to reduce incidents with inpatients resulting in crises, and over time help work towards recovery. In turn, this might help to encourage cognitive participation and collective action in staff implementing the intervention [32]. Indeed, a recent RCT reported that staff training in evidence-based therapeutic activities significantly improved perceptions of and satisfaction with inpatient mental health care in legally detained inpatients [47]. Highlighting the relevance of new interventions may lead to a greater perceived sense of competency in staff [48].

Focus group participants also highlighted accessibility and visibility as key to maintaining the workbook’s use, and suggested putting copies in communal spaces, care plans, welcome packs and on notice boards. This would increase staff and inpatients’ opportunity to engage with the workbook’s content and help to embed its use in the culture of the ward. Its visibility could also act as a prompt to remind staff of the things learnt in training. This suggests that the flexibility and physical accessibility of a workbook format could be particularly helpful in enabling a psychological intervention to become routinely incorporated into the everyday practice of an inpatient ward. Adaptability of interventions has been identified as a key part of a consolidated framework for fostering implementation of health services research findings into practice [49], whilst accessibility and visibility may mediate the link between intending to implement an intervention and actually implementing it [50].

### Strengths and limitations

A strength of the study was the sampling of nursing staff of varying levels of seniority, including unqualified nursing staff, qualified nurses and ward managers, as well as occupational therapists and clinical psychologists. This ensured that the voices of the nursing staff, who would be primarily involved in delivering such an intervention, were prioritised, whilst the multidisciplinary nature of work in inpatient psychiatric wards was acknowledged. This enabled representation of a breadth of perspectives and experiences of working on the wards. The separation of staff in more senior and less senior positions into different focus groups also helped to minimise the impact of hierarchy on the discussions. An additional strength is that much of the feedback from the present investigation might be applicable to other interventions aimed at emotion regulation. We hope that these findings are contemplated during the development of such interventions.

A limitation is that the present investigation was restricted to two NHS hospitals. The findings are not necessarily generalisable to other hospitals around or outside of the UK, which may differ in terms of service structure, access to and distribution of resources, and staff and inpatient demographics. Additionally, it is unclear whether the way staff anticipate use of the workbook on wards, as described in this investigation, will reflect the real outcome. Furthermore, this study did not include the feedback of another crucial stakeholder group, the inpatients themselves. Finally, the study authors consisted of academic and clinical psychologists not currently employed on inpatient wards – this reinforces the vital importance of consulting inpatient psychiatric staff, as we have done, and ultimately service-users, when designing such a resource.

### Implications for further research

The next stage of workbook development will be to incorporate the feedback from this investigation into an updated version of the Emotional Coping Skills workbook. Following this, it will be essential to gain feedback from inpatients and use this to modify the workbook further. If this suggests that the workbook is likely to be feasible and acceptable, it would be useful to pilot it on an inpatient ward to determine whether this remains true in practice, and to generate preliminary evidence on its effectiveness.

## Conclusions

Through analysis of the rich and insightful data generated by focus groups with inpatient psychiatric nursing staff and other relevant stakeholders, the present investigation revealed a number of potential benefits and barriers to using a workbook to help inpatients understand and cope with emotional distress. The feedback will be incorporated into refining and improving the intervention so that it can enter further stages of development. Furthermore, the findings highlight principles that are necessary to consider when developing and implementing psychological interventions on inpatient wards; firstly, the value of creating a resource that can easily be integrated into current nursing practice and compliment nurses’ existing tasks rather than adding to their workload. Secondly, the importance of helping staff to see the usefulness of psychological approaches in a culture dominated by medical ones. Thirdly, the utility of interventions that can be flexibly applied in different formats and modes of delivery, and, finally, the critical importance of enabling nursing staff to use their judgement and expertise to decide the timing of intervention delivery in the context of each inpatient’s fluctuations in distress and progress towards recovery. The investigation provides a promising step in the continued efforts to make the inpatient psychiatric hospital a more therapeutic environment.
